# Exploring the Link Between Genetic Predictors of Cardiovascular Disease and Psoriasis

**DOI:** 10.1001/jamacardio.2024.2859

**Published:** 2024-09-18

**Authors:** Ravi Ramessur, Jake Saklatvala, Ashley Budu-Aggrey, Marek Ostaszewski, Lena Möbus, Dario Greco, Matladi Ndlovu, Satveer K. Mahil, Jonathan N. Barker, Sara Brown, Lavinia Paternoster, Nick Dand, Michael A. Simpson, Catherine H. Smith

**Affiliations:** 1 St John’s Institute of Dermatology, School of Basic & Medical Biosciences, Faculty of Life Sciences & Medicine, King’s College London, London, United Kingdom; 2 Department of Medical and Molecular Genetics, School of Basic & Medical Biosciences, King’s College London, London, United Kingdom; 3 MRC Integrative Epidemiology Unit at University of Bristol, Bristol, United Kingdom; 4 Population Health Sciences, Bristol Medical School, Bristol, United Kingdom; 5 Luxembourg Centre for Systems Biomedicine, University of Luxembourg, Esch-sur-Alzette, Luxembourg; 6 Finnish Hub for Development and Validation of Integrated Approaches, Faculty of Medicine and Health Technology, Tampere University, Tampere, Finland; 7 Division of Pharmaceutical Biosciences, Faculty of Pharmacy, University of Helsinki, Helsinki, Uusimaa, Finland; 8 Department of Immunology Research, UCB, Brussels, Belgium; 9 Centre for Genomic and Experimental Medicine, University of Edinburgh, Edinburgh, Scotland, United Kingdom; 10 Department of Dermatology, NHS Lothian, Edinburgh, Scotland, United Kingdom; 11 NIHR Bristol Biomedical Research Centre, University Hospitals Bristol and Weston NHS Foundation Trust and University of Bristol, Bristol, United Kingdom

## Abstract

**Importance:**

The epidemiological link between immune-mediated diseases (IMIDs) and cardiovascular disease has often been attributed to systemic inflammation. However, the direction of causality and the biological mechanisms linking cardiovascular disease with IMIDs are incompletely understood. Given the robust epidemiological association and the growing body of supportive mechanistic evidence, psoriasis is an exemplary IMID model for exploring this relationship.

**Objective:**

To assess the bidirectional relationships between genetic predictors of psoriasis and the 2 major forms of cardiovascular disease, coronary artery disease (CAD) and stroke, and to evaluate the association between genetic predictors of cardiovascular disease with 9 other IMIDs.

**Design, Setting, and Participants:**

This was a genetic association study using mendelian randomization (MR), a powerful genetic tool to help distinguish causation from associations observed in epidemiological studies, to provide supportive evidence for causality between traits. The study conducted 2-sample MR analyses using summary-level data from large-scale genome-wide association meta-analysis studies (GWAS) for each trait. The analysis focused on individuals of European descent from GWAS meta-analyses, involving CAD, stroke, psoriasis, and 9 other IMIDs. Data were analyzed from January 2023 to May 2024.

**Exposures:**

Genetic predictors of CAD, stroke, psoriasis, and 9 other IMIDs.

**Main Outcomes and Measures:**

The primary outcomes were the associations of genetic predictors of CAD and stroke with the risk of psoriasis and 9 other IMIDs, determined using inverse-variance weighted (IVW) MR estimates.

**Results:**

This study included 181 249 cases and 1 165 690 controls with CAD, 110 182 cases and 1 503 898 controls with stroke, 36 466 cases and 458 078 controls with psoriasis, for a total of approximately 3 400 000 individuals, and 9 other IMIDs. In contrast to previous assumptions, genetic predictors of psoriasis were found to have no association with CAD or stroke. In the reverse direction, genetic predictors of both CAD (MR estimate IVW odds ratio [OR], 1.07; 95% CI, 1.04-1.10; *P* = .003) and stroke (IVW OR, 1.22; 95% CI, 1.05-1.41; *P* = .01) were found to have risk-increasing associations with psoriasis. Adjusting for stroke rendered the associations of genetically predicted CAD with psoriasis risk nonsignificant (and vice versa), suggesting that a shared effect underlying genetic risk for CAD and stroke associates with increased psoriasis risk. No risk-increasing associations were observed for genetic predictors of cardiovascular disease with other common IMIDs, including rheumatoid arthritis and inflammatory bowel disease.

**Conclusions and Relevance:**

Findings of this mendelian randomization study indicate that genetic predictors of cardiovascular disease were associated with increased psoriasis risk with no reciprocal effect or association with other IMIDs. Elucidating mechanisms underpinning this association could lead to novel therapeutic approaches in both diseases.

## Key Points

**Question:**

Does genetic risk for cardiovascular disease predispose individuals to immune-mediated diseases (IMIDs)?

**Findings:**

In this mendelian randomization study using datasets comprising more than 500 000 participants, genetic predictors of coronary artery disease (CAD) and stroke were found to be statistically significantly associated with increased psoriasis risk. In contrast, no statistically significant risk-increasing associations were observed for genetic predictors of CAD or stroke on several other IMIDs, including rheumatoid arthritis and inflammatory bowel disease.

**Meaning:**

These findings suggest that biological pathways influencing cardiovascular disease risk are associated with psoriasis risk but not with other IMIDs.

## Teaser

This mendelian randomization study investigates the association between genetic risk for cardiovascular disease and immune-mediated diseases.

## Introduction

Systemic inflammation plays a significant role in the pathogenesis of cardiovascular disease.^1^ Supporting this concept, anti-inflammatory treatments (including canakinumab and colchicine) have been shown to be effective in reducing the incidence of cardiovascular events in several clinical trials.^2,3,4,5^ Numerous inflammatory biomarkers, including CD14++CD16+ monocytes and high-sensitivity C-reactive protein, predict cardiovascular disease independently of traditional cardiovascular risk factors in high-risk individuals undergoing elective coronary angiography.^6,7^ These observations contribute to the prevailing hypothesis that the epidemiological association between immune-mediated disease (IMID) and cardiovascular disease is driven by systemic inflammation.^8,9,10,11,12,13^ Nevertheless, the direction of causality, the role of key confounding factors, and the biological pathways linking cardiovascular disease with IMIDs remain incompletely understood.^1,14^

Psoriasis serves as an exemplary model for studying this relationship. It is a highly prevalent IMID with a distinct clinical phenotype that rarely necessitates extensive investigation for diagnosis, therefore, it is well captured in large-scale population-based studies.^15^ A well-established, epidemiological association links psoriasis to the 2 major forms of cardiovascular disease: coronary artery disease (CAD) and stroke.^16,17^ Severe psoriasis has been shown to be an independent risk factor in the development of cardiovascular disease^11^ and is now integrated in cardiovascular risk assessment tools for clinical practice.^18^ As with other IMIDs, important cardiovascular risk factors,^19^ obesity, and dyslipidemia, are highly prevalent in the psoriasis population—particularly in those with severe psoriasis where the cardiovascular risk seems to be greatest. Mechanistic evidence also supports the notion that psoriasis-associated systemic inflammation drives this causal relationship: (1) there is overactivation of the interleukin 17/23 canonical pathway central to psoriasis disease pathogenesis, (2) interleukin 17/23 is also found in both the blood and carotid atherosclerotic plaques in people with cardiovascular disease,^20,21^ and (3) targeted blockade improves imaging-based measures of subclinical CAD in individuals with psoriasis. Collectively, these findings have led some to advocate early intervention in psoriasis to abrogate cardiovascular risk.^22,23,24^

Recently, this view has been challenged by a study by Patrick et al, which used mendelian randomization (MR)—a powerful genetic tool to distinguish causation from associations observed in epidemiological studies.^25,26^ This study revealed a risk-increasing association between genetic predictors of CAD on psoriasis, rather than psoriasis on CAD, in contrast with common dogma.^27,28^ Notably, they found that this effect remained significant after adjusting for body mass index and waist to hip ratio. However, considering the numerous shared risk factors between psoriasis and cardiovascular disease, the impact of other potential confounding factors is still unclear. Furthermore, whether these putative causal effects are also observed in other forms of cardiovascular disease or if they play a risk-increasing role on other IMIDs has not, to our knowledge, been investigated. Building on these findings, our study uses the most powerful genetic instruments currently available, from recent large-scale genome-wide meta-analyses,^29,30,31^ thereby maximizing the precision of associations. We assessed the bidirectional relationship between genetic predictors of psoriasis and the 2 major forms of cardiovascular disease: CAD and stroke. We address several important gaps by (1) investigating whether putative associations between genetic predictors of CAD and psoriasis are specific or also shared in stroke (2) evaluating the impact of several key potential confounders, (3) determining whether associations are specific to psoriasis or are generalized to other IMIDs, and (4) exploring the role of commonly prescribed medications for cardiovascular disease as potential mediators of the observed effects.

## Methods

Two-sample MR analyses do not involve human participants, individual patient data, or any interaction or intervention with human individuals. Instead, 2-sample MR exclusively uses genetic variants from previously published genome-wide association studies (GWAS). As such, no ethical approval was required for these analyses. The UK Biobank analyses were carried out using the UK Biobank Resource, approved under project number 15147. The UK Biobank has also obtained clearance from the Patient Information Advisory Group in England and Wales to access data that enables the invitation of potential participants. Written informed consent was required for participant enrollment in the UK Biobank study. This study followed the Strengthening the Reporting of Observational Studies in Epidemiology Using Mendelian Randomization (STROBE-MR) guidelines.^32^ The eMethods in Supplement 1 contains further information on detailed study methodology.

### Study Design and Data Sources

This study uses MR to explore the relationship between cardiovascular disease and psoriasis as well as other common IMIDs. MR is a method that uses genetic variants to help determine if there is a causal relationship between an exposure and an outcome.^25^ By leveraging the fixed nature of genetic variants from conception, MR helps overcome issues of unmeasured confounding and reverse causation found in observational studies, providing supportive evidence for causality.

Two-sample MR with summary-level data from GWAS was used to explore the bidirectional relationship between genetic predictors of psoriasis and cardiovascular disease, including CAD and stroke.^33^ The association of genetic predictors of CAD and stroke with psoriasis was investigated using multivariable MR to adjust for potential confounding factors common to both conditions (body mass index, waist to hip ratio, smoking, systolic blood pressure, diastolic blood pressure, hemoglobin A_1c_, total cholesterol, triglycerides, and low-density lipoprotein cholesterol). Additionally, the association of genetic predictors of cardiovascular disease with 9 other common IMIDs was examined; for these IMIDs, an epidemiological association with cardiovascular disease had been previously reported (ie, acne,^34^ atopic dermatitis,^35^ asthma,^36^ celiac disease,^37^ Crohn disease,^38^ inflammatory bowel disease,^38^ multiple sclerosis,^39^ rheumatoid arthritis,^11^ ulcerative colitis^40^) to reveal the specificity or generality of cardiovascular genetic risk factor associations across IMIDs.

For further in-depth analyses in specific population subgroups, 1-sample MR was performed in the UK Biobank using individual-level data (eTables 1-3 in Supplement 2). Cardiovascular genetic risk factor associations were examined stratified by common medication use, CAD cases (vs controls), sex-specific differences, and HLA-C*06:02-status (primary susceptibility allele in psoriasis) in view of epidemiological evidence of higher prevalence of cardiovascular disease in these subgroups, as detailed in eMethods in Supplement 1. Analyses were restricted to only individuals of White European ancestry to maximize the consistency and reliability of genetic instruments used.

A psoriasis genetic instrument (109 independent variants genome-wide significant variants [*P* value <5 × 10^−08^]), was derived from a psoriasis GWAS meta-analysis.^30^ CAD (241 independent variants) and stroke (47 independent variants) genetic instruments were derived from genome-wide significant variants from CAD^31^ and stroke^29^ GWAS meta-analyses, respectively (eTables 4-6 in Supplement 2). These genetic instruments were used in both 1- and 2-sample MR. For other IMIDs, data from the following GWAS meta-analyses were used: acne,^41^ atopic dermatitis,^42^ asthma,^43^ celiac disease,^44^ Crohn disease,^45^ inflammatory bowel disease,^45^ multiple sclerosis,^46^ rheumatoid arthritis,^47^ or ulcerative colitis.^45^

### Assumptions

A genetic variant can be considered as an instrumental variable for a given exposure if it satisfies the instrumental variable assumptions: (1) it is associated with the exposure, (2) it is not associated with the outcome through confounding pathways, and (3) it does not affect the outcome except potentially via the exposure.

### Statistical Analysis

The primary statistical method for evaluating MR effects was the inverse-variance weighted (IVW) MR. For each outcome, we reported the estimated effect of the binary exposure variable as an odds ratio (OR) with corresponding 95% CIs. The association of genetic predictors of cardiovascular disease with the 9 other IMIDs is presented with 1-tailed *P* values to test the hypothesis that genetic predictors of cardiovascular disease have risk-increasing associations with IMIDs. All other *P* values are presented with 2-tailed *P* values as measures of statistical significance. A schematic representation of bidirectional MR analyses is available in eFigure 1 in Supplement 1.

#### Assessment of Assumptions

The *F* statistic assumptions in 2-sample MR were as follows: 150.4 for psoriasis, 73.8 for CAD, and 44.3 for stroke. All genetic instruments used had an *F* statistic of 10 or greater, indicating sufficient instrument strength to avoid weak instrument bias.^48^

#### Sensitivity Analyses

Results have potential for bias if the instrumental variables exhibit horizontal pleiotropy, influencing the outcome through causal pathways other than the exposure, thus violating the third instrumental variable assumption.^49^ To address this, we performed sensitivity analyses using MR-Egger, simple median, and weighted median on all analyses.^50^ For 2-sample MR analyses examining the bidirectional relationship between genetic predictors of psoriasis and cardiovascular disease, MR-Steiger sensitivity testing was also performed to test whether the assumption that the exposure causes the outcome is valid^51^ Additionally, MR-Lasso and MR-PRESSO sensitivity analyses^52^ were performed to examine the effects of removing outliers, and the Cochran *Q* statistic was calculated to establish the presence of heterogeneity.^52,53^ Scatterplots, funnel plots, and leave-one-out forest plots for the main 2-sample MR effects are presented in eFigure 2 in Supplement 1. All MR analyses were performed using R, version 4.1.2 (R Foundation for Statistical Computing) using the TwoSampleMR, MendelianRandomization, and MVMR packages.^53,54^ Data were analyzed from January 2023 to May 2024.

## Results

### Genetic Risk for Stroke/CAD and Psoriasis Risk

Data from a total of approximately 3 400 000 individuals were included in this analysis. A psoriasis genetic instrument was derived from a psoriasis GWAS meta-analysis (36 466 cases; 458 078 controls). CAD (241 independent variants) and stroke (47 independent variants) genetic instruments were derived from genome-wide significant variants from CAD (181 249 cases; 1 165 690 controls) and stroke (110 182 cases; 1 503 898 controls) GWAS meta-analyses, respectively (eTables 4-6 in Supplement 2 and eAppendix in Supplement 3). These genetic instruments were used in both 1- and 2-sample MR. For other IMIDs, data from the following GWAS meta-analyses were used: acne (20 165 cases; 595 231 controls),^41^ atopic dermatitis (60 653 cases; 804 329 controls),^42^ asthma (19 954 cases; 107 715 controls),^43^ celiac disease (4533 cases; 10 750 controls),^44^ Crohn disease (40 266 cases; 28 072 controls),^45^ inflammatory bowel disease (59 957 cases; 34 915 controls),^45^ multiple sclerosis (47 429 cases; 68 374 controls),^46^ rheumatoid arthritis (29 880 cases; 73 758 controls),^47^ or ulcerative colitis (33 609 cases; 45 975 controls).^45^

No statistically significant associations were observed for genetic predictors of psoriasis with CAD (IVW OR, 1.01; 95% CI, 0.99-1.03; *P* = .12) or stroke (IVW OR, 1.00; 95% CI, 0.98-1.02; *P* = .77) (Table 1 and eTable 7 in Supplement 2).

**Table 1.** Two-Sample Mendelian Randomization Examining the Bidirectional Relationship Between Genetic Predictors of Psoriasis and Coronary Artery Disease (CAD)/Stroke^a^

Exposure	Outcome	Unadjusted model OR (95% CI)	Unadjusted model *P* value	CAD-adjusted OR (95% CI)	CAD-adjusted *P* value	Stroke-adjusted OR (95% CI)	Stroke-adjusted *P* value	Confounder-adjusted model OR (95% CI)^b^	Confounder-adjusted model *P* value^b^
Psoriasis (109 variants)	CAD	1.01 (0.99-1.03)	.12	NA	NA	NA	NA	NA	NA
Psoriasis (109 variants)	Stroke	1.00 (0.98-1.02)	.77	NA	NA	NA	NA	NA	NA
CAD (234 variants)	Psoriasis	1.07 (1.04-1.10)^c^	.003^c^	1.04 (0.98-1.09)	.21	NA	NA	1.06 (1.01-1.11)^c^	.02^c^
Stroke (47 variants)	Psoriasis	1.22 (1.05-1.41)^c^	.01^c^	NA	NA	1.14 (0.97-1.35)	.12	1.17 (1.05-1.29)^c^	.02^c^

Abbreviations: NA, not applicable; OR, odds ratio.

^a^ Adjustments for stroke/CAD effects and key confounders assessed using multivariable MR.

^b^ Key confounders included collectively in a multivariable mendelian randomization model: body mass index, waist to hip ratio, smoking, systolic blood pressure, diastolic blood pressure, hemoglobin A_1c_, total cholesterol, triglycerides, and low-density lipoprotein cholesterol.

^c^ Statistically significant (*P* < .05) results.

A significant association was found between genetic predictors of CAD and psoriasis (IVW OR, 1.07; 95% CI, 1.04-1.10; *P* = .003). We also report an association between genetic predictors of stroke and psoriasis (IVW OR, 1.22; 95% CI, 1.05-1.41; *P* = .01) (Table 1 and eTable 7 in Supplement 2). No statistically significant difference between MR estimates for the effect of genetic predictors of CAD or stroke on psoriasis was observed. MR-Steiger directionality testing was conducted to ensure the directionality of the instrument was for the exposure rather than the outcome, confirming statistically significant risk-increasing MR associations (MR-Steiger MR effect *P* value for genetic predictors of CAD on psoriasis: 1 × 10^−27^ and genetic predictors of stroke on psoriasis: 1 × 10^−32^), increasing confidence in the reported associations.^51^ Statistically significant MR effects were found for genetic predictors of CAD/stroke on psoriasis in simple median, weighted median, MR-PRESSO, and MR-Lasso sensitivity analyses, but no statistically significant MR effects were found in MR-Egger analyses (eTable 7 in Supplement 2).

To determine whether the MR effects of genetic predictors of CAD and stroke were shared, multivariable MR was conducted, adjusting for the effects of the alternative cardiovascular trait. The MR effect of genetic predictors of CAD on psoriasis was no longer statistically significant after adjusting for stroke (CAD IVW MR estimate after adjusting for the stroke effect: OR, 1.04; 95% CI, 0.98-1.09; *P* = .21). Equally, the MR effect of genetic predictors of stroke on psoriasis was no longer statistically significant after adjusting for CAD (stroke IVW MR estimate after adjusting for the CAD effect: OR, 1.14; 95% CI, 0.97-1.35; *P* = .12) (Table 1). These findings indicate a substantial overlap in these MR effects, suggesting that a shared cardiovascular effect underlying genetic risk for CAD and stroke was associated with an increased risk of psoriasis.

To assess the independence of MR effects from key confounding factors, multivariable MR was performed. Including 9 key confounding factors in a multivariable model, individually (eTable 9 in Supplement 2) or collectively (Table 1), the MR effects remained statistically significant (CAD IVW estimate after inclusion of all key confounders: OR, 1.06; 95% CI, 1.01-1.11; *P* = .02; stroke IVW estimate after inclusion of all key confounders: OR, 1.17; 95% CI, 1.05-1.29; *P* = .02). These findings support the independence of the observed cardiovascular MR effects from key confounding factors.

For further in-depth analyses in specific subgroups of the population, 1-sample MR was performed in the UK Biobank. The direction of associations between genetic risk for CAD, stroke, and psoriasis was found to be consistent with the findings in the 2-sample MR analysis. However, the association between genetic predictors of CAD with psoriasis risk was not statistically significant (IVW OR, 1.04; 95% CI, 0.95-1.14; *P* = .40), whereas the association between genetic predictors of stroke with psoriasis risk was statistically significant (IVW OR, 1.27; 95% CI, 1.06-1.50; *P* = .03) (Table 2; and eTable 8 in Supplement 2). Aligned with the 2-sample MR results, no statistically significant associations were observed for genetic predictors of psoriasis with CAD (IVW OR, 1.01; 95% CI, 0.99-1.03; *P* = .11) or stroke (IVW OR, 1.01; 95% CI, 0.98-1.04; *P* = .58) in the 1-sample MR analysis in the UK Biobank. We then explored the presence of sex-specific and HLA-C*06:02 subgroup–specific differences in MR effects given the higher prevalence of cardiovascular disease in females (vs males) with psoriasis^55,56^ and in those who have no copies HLA-C*06:02 allele (vs participants carrying 1 or 2 copies of the HLA-C*06:02 allele). No difference in CAD or stroke MR effects on psoriasis were observed in sex-stratified or HLA-C*06:02-stratified subgroups (eTables 10 and 11 in Supplement 2).

**Table 2.** One-Sample Mendelian Randomization (MR) Examining the Bidirectional Relationship Between Genetic Predictors of Psoriasis and Coronary Artery Disease (CAD)/Stroke^a^

Exposure	Outcome	UK Biobank					
		Full cohort (n = 336 806)		CAD cases (n = 33 466)		CAD controls (n = 303 340)	
		OR (95% CI)	*P* value	OR (95% CI)	*P* value	OR (95% CI)	*P* value
Psoriasis (109 variants)	CAD	1.01 (0.99-1.03)	.11	NA	NA	NA	NA
Psoriasis (109 variants)	Stroke	1.01 (0.98-1.04)	.58	NA	NA	NA	NA
CAD (234 variants)	Psoriasis	1.04 (0.95-1.14)	.40	0.86 (0.67-1.12)	.14	1.02 (0.93-1.12)	.66
Stroke (47 variants)	Psoriasis	1.27 (1.06-1.50)^b^	.03^b^	0.84 (0.53-1.32)	.71	1.10 (0.91-1.32)	.66

Abbreviations: NA, not applicable; OR, odds ratio.

^a^ One-sample MR was performed using individual level data on an unrelated White British subset of UK Biobank participants. CAD (33 466 cases) and stroke (10 593 cases) cases were indicated by participant self-report at baseline assessment and/or a primary or secondary diagnosis in linked in-patient hospital episode statistics and/or death registry diagnosis and/or relevant procedure codes and/or primary care diagnosis from linked primary care data. Stratification by CAD status performed to investigate whether commonly prescribed CAD medications mediate observed cardiovascular MR associations.

^b^ Statistically significant (*P* < .05) results.

### Cardiovascular MR Effects and Other Common IMIDs

Having established evidence of an association between genetic predictors of cardiovascular traits and psoriasis, we then investigated whether risk-increasing cardiovascular MR associations were observed in other common IMIDs. No risk-increasing associations were observed for genetic predictors of CAD or stroke with acne, atopic dermatitis, asthma, celiac disease, Crohn disease, inflammatory bowel disease, multiple sclerosis, rheumatoid arthritis, or ulcerative colitis (Table 3; and eTable 14 in Supplement 2).

**Table 3.** Two-Sample Mendelian Randomization (MR) Examining for Risk-Increasing MR Associations Between Cardiovascular Traits and Common Immune-Mediated Diseases

Outcome (immune-mediated disease)	Coronary artery disease (exposure) [234 variants]		Stroke (exposure) [47 variants]	
	OR (95%CI)	One-tailed *P* value	OR (95% CI)	One-tailed *P* value
Atopic dermatitis	1.03 (0.96-1.10)	.18	0.91 (0.77-1.08)	.86
Acne	0.96 (0.91-1.00)	.97	0.92 (0.77-1.09)	.85
Asthma	0.97 (0.94-1.01)	.92	0.87 (0.80-0.94)	>.99
Multiple sclerosis	1.06 (0.94-1.19)	.17	0.83 (0.66-1.04)	.95
Celiac disease	1.00 (0.99-1.01)	.36	0.99 (0.98-1.02)	.60
Rheumatoid arthritis	0.92 (0.84-1.01)	.95	1.00 (0.81-1.23)	.48
Inflammatory bowel disease	0.97 (0.91-1.04)	.80	0.95 (0.81-1.13)	.71
Crohn disease	0.95 (0.88-1.04)	.87	1.00 (0.81-1.24)	.49
Ulcerative colitis	0.99 (0.93-1.06)	.58	0.92 (0.79-1.09)	.83

Abbreviation: OR, odds ratio.

### Common Medications for Cardiovascular Disease and Psoriasis

To investigate whether commonly prescribed medications for cardiovascular disease mediate the observed cardiovascular MR effects, 1-sample MR was conducted in the UK Biobank, stratifying for CAD cases and controls. The associations of genetic predictors of CAD or stroke with psoriasis risk were not significantly different in CAD cases (CAD IVW OR, 0.86; 95% CI, 0.67-1.12; *P* = .14; stroke IVW OR, 0.84; 95% CI, 0.53-1.32; *P* = .71) compared with CAD controls (CAD IVW OR, 1.02; 95% CI, 0.93-1.12; *P* = .66; stroke IVW OR, 1.10; 95% CI, 0.91-1.32; *P* = .66) (Table 2; eTable 12 in Supplement 2). Cardiovascular MR effects on psoriasis were not stronger in participants taking β-blockers, angiotensin-converting enzyme inhibitors, angiotensin receptor blockers, aspirin, or statins, compared with participants who did not take these medications (eTable 13 in Supplement 2). These findings collectively suggest that commonly prescribed medications for cardiovascular disease are unlikely to mediate the observed cardiovascular MR effects.

## Discussion

Our findings are consistent with genetic predictors of cardiovascular disease associating with increased psoriasis risk with no reciprocal effect. This suggests that pathways influencing cardiovascular disease risk may also contribute to the causal biological pathways of psoriasis. Risk-increasing associations were not observed between cardiovascular disease and other common IMIDs, indicating that these associations may be specific to the psoriatic disease context. These findings, therefore, provide new insights into the pathogenesis of both psoriasis and cardiovascular disease.

This study applies the most powerful genetic instruments for psoriasis, stroke, and CAD to date to examine the bidirectional relationship between genetic risk for psoriasis and cardiovascular disease, maximizing the precision of associations. We found an association between genetic predictors of stroke and psoriasis that, to our knowledge, has not been previously reported. In line with Patrick et al,^26^ we found a risk-increasing association between genetic predictors of CAD and psoriasis. Multivariable MR supported a shared cardiovascular effect underlying genetic risk for stroke and CAD that is likely to mediate the observed MR effects. The MR effect point estimate was larger for genetic predictors of stroke compared with CAD on psoriasis in both 1-sample and 2-sample MR approaches used in our study. Our stroke findings are consistent with those of Ogdie et al^11^ reporting a comparatively stronger epidemiological association between psoriasis and stroke (compared with psoriasis and heart disease) in a large population-based longitudinal study. This may indicate that the underlying cardiovascular MR effect with psoriasis may be more strongly represented in the stroke compared with the CAD genetic instrument. Dissecting the genetic instruments to establish shared stroke and CAD susceptibility variants that drive this MR effect on psoriasis is an important future direction to help investigate which aspects of the shared biology contribute to increased psoriasis risk.

In MR analyses involving binary or coarsened exposures, interpretations should consider the liability threshold model. This model posits that genetic variants impact a continuous underlying risk that, when a threshold is crossed, manifests as a binary outcome (eg, disease presence or absence). Our genetic instruments for the lifetime risk of cardiovascular disease and psoriasis are reflective of this continuous risk, not merely a binary state. Supported by recent studies,^57,58^ this approach underscores that our findings indicate a shared predisposition for both conditions, highlighting their biological linkage.

These findings challenge 2 prevailing concepts regarding the link between psoriasis and cardiovascular disease. First, the strong association between psoriasis and cardiovascular disease is attributable to the high prevalence of cardiovascular risk factors in psoriasis. Many of these risk factors (eg, increasing levels of adiposity) are part of the composite diagnosis of metabolic syndrome and are known to be risk factors for psoriasis.^59,60^ Our findings suggest that a cardiovascular MR effect on psoriasis may account for some of the epidemiological association observed, and these effects may be independent of direct effects of several cardiovascular risk factors. Second, these findings challenge the common dogma that inflammation from psoriasis exerts a systemic inflammatory effect that predisposes individuals with psoriasis to cardiovascular disease. Although associations with small effect sizes cannot be entirely ruled out due to inherent limitations in instrument strength and power, our study found no evidence that genetic predictors of psoriasis were associated with CAD or stroke in either 1-sample or 2-sample MR analyses.

The use of genetic instruments to evaluate causal relationships represents lifetime risk of exposure development and, thus, provides a measure of predisposition to an outcome over a lifetime. Our findings may at first seem at odds with epidemiological studies showing an increased risk of incident cardiovascular disease in people with psoriasis and that, generally, cardiovascular disease tends to manifest later in life when compared with psoriasis onset.^61^ However, the processes driving disease development and the clinical manifestation of disease are related but temporally distinct. Several studies have demonstrated the presence of progression toward pathological vascular changes from early adulthood in postmortem examinations from younger adults who died of natural causes,^62,63^ demonstrating that subclinical cardiovascular disease is typically present for decades before clinical symptoms and signs. Our findings are thus consistent with the notion that the underlying mechanisms contributing to these cumulative changes are also associated with increased psoriasis risk.

In this study, we conducted an ancestry-specific analysis in Europeans. The strength of MR associations in other ancestral groups is unknown and will be subject to future work. Given the substantially higher reported prevalence of cardiometabolic comorbidity in severe psoriasis compared with nonsevere disease,^11,16,17^ it is possible that the strength of MR associations may also vary between subgroups defined by psoriasis severity.

Our findings also point to surprising potential differences in the biology of common IMIDs. Many IMIDs share underlying immune mechanisms^64^; however, differences also exist. For example, the role of helper T cells in inflammation is prominent in atopic diseases such as asthma and atopic dermatitis,^65^ whereas B cell–mediated autoantibodies play a significant role in rheumatoid arthritis.^66^ Our findings suggest that the influence of biological pathways underpinning cardiovascular disease may not have the same effect on predisposition to all common IMIDs. Given that risk-increasing MR associations were not observed for genetic predictors of CAD or stroke on several other common IMIDs, an MR effect in the reverse direction could explain reported epidemiological associations.^67,68,69^ Relationships between genetic predictors of other IMIDs on cardiovascular disease, as well as the role of potential confounding factors such as smoking and obesity, are important to investigate in future studies.

Although our study was unable to establish specific mechanisms underlying cardiovascular MR associations, we did not find evidence of commonly prescribed medications for cardiovascular disease being mediators of the effects observed. Future work should focus on understanding the mechanisms underpinning these cardiovascular MR associations through dissection of the stroke and CAD genetics instruments, including distinguishing the effects of CAD/stroke genes with known roles in systemic inflammation. Our findings may have important implications for the management and prevention of cardiovascular disease in patients with psoriasis. By providing supportive evidence for an association between genetic predictors of cardiovascular disease and increased psoriasis risk, our findings could suggest that vigilance in monitoring cardiovascular health and implementing early interventions (eg, lifestyle modifications and pharmacotherapy) to reduce the risk of cardiovascular disease people with psoriasis may also aid psoriasis management. Moreover, the identification of shared genetic pathways influencing both psoriasis and cardiovascular disease provide a deeper understanding of the pathophysiology underlying these conditions. This knowledge could help expedite biomarker discovery to improve cardiovascular risk prediction in the psoriasis population and lead to the development of novel therapeutic strategies that target common inflammatory pathways, potentially offering dual benefits in treating psoriasis and preventing cardiovascular disease.

### Limitations

This study’s limitations include the inability to empirically prove that the second and third instrumental variable assumptions have not been violated in MR analyses, as this relies on investigator judgment.^70^ Although bidirectional MR provides valuable insights into reciprocal causal relationships between traits, it carries additional assumptions and limitations beyond those of standard unidirectional MR. First, bidirectional MR requires strong, independent genetic instruments free from pleiotropic effects. Weak instruments for either trait can bias the estimates and reduce the power of the bidirectional MR analysis. However, the use of the most powerful genetic instruments currently available helped minimize the impact of this limitation in our study. Second, overlapping cohorts for estimating the genetic associations with both traits can introduce bias due to sample overlap, leading to inflated type I error rates and biased causal estimates. For the main 2-sample MR effects in our study, the results remained significant when removing overlapping cohorts from the psoriasis GWAS summary data (eTable 15 in Supplement 2). Although our study had sufficient power to detect moderate to large effects, it may have been underpowered to identify smaller effect sizes between genetic predictors of cardiovascular disease and common IMIDs such as celiac disease and multiples sclerosis (eTable 16 in Supplement 1 and eTable 16 in Supplement 2). Future studies with larger sample sizes or more powerful genetic instruments are required to conclusively address these relationships.

## Conclusions

In summary, findings from this mendelian randomization study suggest that genetic predictors of cardiovascular disease were associated with increased psoriasis risk with no reciprocal effect or association with other IMIDs. Considering growing evidence for the role of inflammation in cardiovascular pathogenesis^1,4,6,7,14,71^ and the known influential role of inflammatory cellular and cytokine pathways underpinning psoriasis,^72^ the shared mechanism linking these traits may have an inflammatory component. Future studies should focus on characterizing the mechanism underlying this cardiovascular effect.

## Notes

Supplement 1. **eMethods.** Detailed Methodology **eFigure 1.** Schematic Representation of Bidirectional Mendelian Randomization (MR) Analyses **eFigure 2.** Plots for 2-Sample Mendelian Randomization
eReferences

Supplement 2. **eTable 1.** UK Biobank Codes for Coronary Artery Disease Case (CAD) Definition **eTable 2.** UK Biobank Codes for Stroke Case Definition **eTable 3.** UK Biobank Prescription Codes for Common Medications for Cardiovascular Disease **eTable 4.** CAD Genetic Variants for Mendelian Randomization **eTable 5.** Stroke Genetic Variants for Mendelian Randomization **eTable 6.** Psoriasis Genetic Variants for Mendelian Randomization **eTable 7.** Two-Sample Bidirectional Mendelian Randomization Examining the Relationship Between Psoriasis and CAD/Stroke **eTable 8.** One-Sample Bidirectional Mendelian Randomization Examining the Relationship Between Psoriasis and CAD/Stroke **eTable 9.** Two-Sample Multivariable Mendelian Randomization Examining the Relationship Between CAD/Stroke and Psoriasis Adjusted for Key Confounders **eTable 10.** Sex-Stratified 1-Sample Mendelian Randomization Examining the Relationship Between CAD/Stroke and Psoriasis **eTable 11.** HLA-C*06:02-Stratified 1-Sample Mendelian Randomization Examining the Relationship Between CAD/Stroke and Psoriasis **eTable 12.** One-Sample Mendelian Randomization Examining the Relationship Between CAD/Stroke and Psoriasis Stratified by CAD Cases/Controls **eTable 13.** One-Sample Mendelian Randomization Examining the Relationship Between CAD/Stroke and Psoriasis Stratified by History of Medication Use **eTable 14.** Two-Sample Mendelian Randomization Examining the Relationship Between CAD/Stroke and Other Common Immune-Mediated Diseases **eTable 15.** Two-Sample Bidirectional Mendelian Randomization Examining the Relationship Between Psoriasis and CAD/Stroke (HUNT, Estonian Biobank and UK Biobank Cohorts Removed From Psoriasis Summary Statistics) **eTable 16.** Power Calculations for 2-sample MR Analyses

Supplement 3.
eAppendix.

Supplement 4.
Data Sharing Statement.
