# Supplementary material for: Exploring the Link Between Genetic Predictors of Cardiovascular Disease and Psoriasis
Source: JAMA Cardiol. 2024 Sep 18;9(11):1009–17. doi: 10.1001/jamacardio.2024.2859 (PMC11411451; doi:10.1001/jamacardio.2024.2859)
Supplement: Supplement 1. — eMethods. Detailed Methodology eFigure 1. Schematic Representation of Bidirectional Mendelian Randomization (MR) Analyses eFigure 2. Plots for 2-Sample Mendelian Randomization eReferences [file jamacardiol-e242859-s001.pdf]

Supplementary Online Content

Ramessur R, Saklatvala J, Budu-Aggrey A, et al. Exploring the link between genetic predictors of cardiovascular disease and psoriasis. *JAMACardiol*. Published online September 18, 2024. doi:10.1001/jamacardio.2024.2859

eMethods. Detailed Methodology

eFigure 1. Schematic Representation of Bidirectional Mendelian Randomization (MR) Analyses

eFigure 2. Plots for 2-Sample Mendelian Randomization

eReferences

This supplementary material has been provided by the authors to give readers additional information about their work.

© 2024 Ramessur R et al. *JAMA Cardiology.*

eMethods. Detailed Methodology

Study design overview

This study uses MR to explore the relationship between cardiovascular disease and psoriasis as well as other immune-mediated diseases (IMIDs) (Figure 1).

Mendelian Randomization (MR) is a powerful statistical method used to assess causality and make inferences about the potential causal relationships between an exposure (or risk factor) and an outcome of interest[1]. MR is frequently used to determine whether observed epidemiological associations are likely to be causal. MR achieves this by leveraging genetic variants, typically single nucleotide polymorphisms (SNPs), as instrumental variables. At conception, genetic alleles are fixed and randomly allocated. This unique characteristic allows MR to circumvent common limitations of observational studies, such as confounding and reverse causation, providing a robust method for estimating the causal effect of a risk factor on an outcome of interest. A genetic variant can be considered as an instrumental variable for a given exposure if it satisfies the instrumental variable assumptions: 1) it is associated with the exposure, 2) it is not associated with the outcome through confounding pathways, and 3) it does not affect the outcome except potentially via the exposure.

MR can be performed using data from a single sample, known as one-sample MR, or from two samples, referred to as two-sample MR. In one-sample MR, genetic variants, exposure, and outcome data are collected from the same individuals, enabling direct comparisons between MR findings and epidemiological associations. In two-sample MR, variant-exposure associations are estimated in one dataset, and variant-outcome associations are estimated in a second dataset, thereby maximising statistical power. Two-sample MR involves datasets from potentially different populations, introducing complexities if the associations of the genetic variants with the exposure or variables on pleiotropic pathways differ between the two samples (creating the potential to violate the validity of the instrumental variable assumptions). This issue becomes particularly relevant when working with diverse ethnic groups, as patterns of linkage disequilibrium may vary between populations, potentially weakening the association between the genetic variant and the exposure in the outcome dataset. For in-depth analyses in specific subgroups or strata of the population, individual-level data is necessary for MR. All of the following analyses were restricted to only individuals of European ancestry to minimise bias from population stratification.

1. Two-sample MR using summary-level data from published genome-wide association study (GWAS) meta-analyses to examine the bidirectional relationship between psoriasis and cardiovascular disease

To evaluate the relationship between psoriasis and cardiovascular disease, the effects of a psoriasis genetic instrument were evaluated in summary statistics from coronary artery disease (CAD) and stroke GWAS meta-analyses[2,3]. The effects of CAD and stroke genetic instruments were then evaluated in the psoriasis GWAS meta-analysis summary statistics[4] to examine the relationship between cardiovascular disease and psoriasis.

Subsequently, to establish whether observed effects were independent of each other, multivariable MR was used to adjust for the effects of the alternate cardiovascular trait. Whilst MR is generally robust to confounding, to exclude confounding effects from well-established shared risk factors for both psoriasis and cardiovascular disease (see 'data sources' below), nine key confounding traits individually and collectively were included in multivariable MR models to assess the independence of MR associations from key confounders.

2. Two-sample MR using summary-level data from published GWASs to examine the relationship between cardiovascular disease and common IMIDs

To investigate whether risk-increasing cardiovascular MR associations were a generalised feature in IMIDs, the CAD and stroke MR associations were examined in nine common IMIDs using two-sample MR. The following nine IMIDs were selected based on the presence of epidemiological evidence of association with cardiovascular disease: acne[5], atopic dermatitis[6], asthma[7], coeliac disease[8], Crohn's disease[9], inflammatory bowel disease[9], multiple sclerosis[10], rheumatoid arthritis[11], or ulcerative colitis[12].

3. One-sample MR using individual-level data in the UK Biobank to explore differences in cardiovascular MR associations stratified by key subgroups

For further in-depth analyses in specific subgroups of the population, one-sample MR using individual-level data from the UK Biobank was performed. Firstly, bidirectional one-sample MR was performed using

individual-level data in the White British subset of the cohort. Cardiovascular MR associations were then examined in key subgroups to a) establish whether CAD medications may be mediators of MR associations observed (examined by comparing MR associations between CAD cases and CAD controls) b) establish whether specific commonly prescribed medications may be mediators of MR associations observed (examined by comparing MR associations between participants with a history of taking commonly prescribed medications for cardiovascular disease prevention [see below] versus participants with no history of taking a given medication) c) establish whether sex-specific difference in MR associations exist in view of the higher reported prevalence of cardiovascular disease in females compared with males with psoriasis[13,14] (examined by comparing MR associations between females versus males) d) establish whether differences between *HLA-C*06:02*-subgroups exists considering evidence indicating a greater burden of cardiovascular comorbidities in people with psoriasis who carry no copies of the primary susceptibility allele in psoriasis *HLA-C*06:02*[15](examined by comparing participants with no copies of *HLA-C*06:02* [*HLA-C*06:02*-negative] versus participants carrying one or two copies of *HLA-C*06:02* [*HLA-C*06:02*-positive]).

Data sources, genetic instruments and study populations

A psoriasis genetic instrument, comprising of 109 independent genome-wide significant variants (p-value< $5x10^{-08}$) was derived from a recent psoriasis GWAS meta-analysis (36,466 cases; 458,078 controls)[4]. CAD and stroke genetic instruments were derived from genome-wide significant variants from large CAD (181,249 cases; 1,165,690 controls)[2] and stroke (110,182 cases; 1,503,898 controls)[3] GWAS meta-analyses respectively. The CAD genetic instrument comprised of 241 independent variants and the stroke genetic instrument had 47 independent variants. These genetic instruments were used in both one- and two-sample MR. If genetic variants were not present in the outcome datasets (summary-level data or UK Biobank) and no suitable proxy variant could be identified, the genetic variant was omitted from MR analysis.

To establish whether MR associations were independent of important confounding factors, key potential confounding factors were selected based on the presence of epidemiological evidence of association with cardiovascular disease and psoriasis, following a literature search as highlighted in step 1 above. The effects of the CAD and stroke genetic instruments on psoriasis were controlled for key potential confounding factors in multivariable MR models. Summary-level data for use in multivariable MR for nine key confounding factors were obtained from their respective GWAS meta-analyses: body mass index (BMI)[16], waist-to-hip ratio (WHR)[16], smoking[17], systolic blood pressure (SBP)[18], diastolic blood pressure (SBP)[18], Hba1c[19], total cholesterol (TC)[20], triglycerides (TG)[20] and LDL cholesterol[20].

The UK Biobank is a population-based prospective study of 502,682 volunteer participants (336,806 participants who were determined to be of White British genetic ancestry by the UK Biobank core team[21]) with biological sample collection and longitudinal follow-up[22]. Participants aged 40–69 years were recruited in 2006–2010. It includes genotypic data, a wide range of detailed phenotypic data, and linkage to electronic health records. Linked primary care data including prescription data is available on a subset of the UK Biobank cohort (147,033 of White British ancestry). Genetic instruments for psoriasis, CAD and stroke were derived from their respective GWAS meta-analyses for one-sample MR in individual-level data in the UK Biobank.

In step 2 (as outlined above), two-sample MR was conducted to examine the effects of the CAD and stroke genetic instruments on nine common IMIDs using the following summary statistics from GWAS meta-analyses: acne (20,165 cases; 595,231 controls)[23], atopic dermatitis (60,653 cases; 804,329 controls)[24], asthma (19,954 cases; 107,715 controls)[25], coeliac disease (4,533 cases; 10,750 controls)[26], Crohn's disease (40,266 cases; 28,072 controls)[27], inflammatory bowel disease (59,957 cases; 34,915 controls)[27], multiple sclerosis (47,429 cases; 68,374 controls)[28], rheumatoid arthritis (29,880 cases; 73,758 controls)[29], or ulcerative colitis (33,609 cases; 45,975 controls)[27].

Genotyping

Genome-wide genotyping array data were generated for UK Biobank participants by Affymetrix using the Applied Biosystems UK BiLEVE Axiom Array or the Applied Biosystems UK Biobank Axiom Array (Applied Biosystems, Waltham, MA), as described elsewhere[21]. Additional quality control steps were applied using PLINK[30]: cases were excluded on the basis of discordant sex information, genotyping rate <99%, relatedness (identity by descent >0.1875), excess heterozygosity rate (mean ± 3 SD), or non-European ancestry. Variants (for use in the calculation of ancestry principal components) were excluded on the basis of missing genotyping rate >1%, minor allele frequency <1%, or departure from Hardy–Weinberg equilibrium ($P < 1 \times 10^{-5}$).

© 2024 Ramessur R et al. *JAMA Cardiology.*

HLA alleles were imputed centrally by the UK Biobank core team using the *HLA*IMP:02* algorithm with a multi-population reference panel[31] for UK Biobank participants. *HLA-C*06:02* status was inferred as negative (zero copies) or positive (one or two copies) where the imputed allele dosage was exactly 0, 1, or 2; participants with imprecise imputed allele count were excluded.

Clinical outcomes and variables

All non-genetic variables in the UK Biobank analysis were ascertained at UK Biobank assessment centre visits. Psoriasis (5,194 cases of White British ancestry) was indicated by participant self-report at baseline assessment (through touchscreen questionnaire and structured interview) and/or a primary or secondary diagnosis in linked in-patient hospital episode statistics (International Classification of Diseases, Revision 10 codes L400, L404, L408, and L409, chosen to best reflect a diagnosis of plaque psoriasis). CAD (33,466 cases of White British ancestry) and stroke (10,593 cases of White British ancestry) cases were indicated by participant self-report at baseline assessment and/or a primary or secondary diagnosis in linked in-patient hospital episode statistics and/or death registry diagnosis and/or relevant procedure codes and/ or primary care diagnosis from linked primary care data. Full lists of codes used for CAD and stroke case definition are listed in Appendix S2.

Several medications that are commonly prescribed for primary or secondary prevention of cardiovascular disease have been reported to be triggers of psoriasis[32]. These include beta blockers[32], ACE inhibitors[32], angiotensin receptor blockers[33], aspirin[32], and statins[34]. Using linked primary care prescription data in the White British subset of the UK Biobank (n=157,022), participants with records of previous prescription of the aforementioned medications were identified for stratified MR analyses. Primary care prescription codes used and the prescription rates in CAD cases/ controls are shown in Appendix S2.

Statistical analysis

All MR analyses were performed using R (version 4.1.2) (R Foundation for Statistical Computing, Vienna, Austria) using the "TwoSampleMR" and "MVMR" packages[35,36].

Data harmonization was carried out to ensure allele correspondence between the exposure and the outcome for two-sample MR analyses. In both one-sample and two-sample MR, all genetic instruments used had an F-statistic of ≥ 10, indicating sufficient instrument strength to avoid weak instrument bias[37].

One-sample MR analysis was performed using the two-stage predictor substitution (TSPS) method[38]. The first stage involved regression of the exposure upon individual genetic variants for the exposure. The outcome was then regressed upon the fitted values from the first regression stage. Because outcomes were binary, the first-stage linear regression was restricted to individuals who were controls for the outcome only, as recommended by Burgess *et al*[39]. Logistic regression was then performed in the second stage, in which the fitted values for the cases were predicted. Inverse-variance weighted MR estimates were obtained from a fixed effects meta-analysis of genetic variant-specific TSPS estimates.

The primary statistical method for evaluating MR associations was the Inverse-variance weighted (IVW) MR. For each outcome, we report the estimated effect of the binary exposure variable as an OR with the corresponding 95% confidence interval. Results have potential for bias if instrumental variables exhibit horizontal pleiotropy, influencing the outcome through causal pathways other than the exposure, thus violating the instrumental variable assumptions as above[40]. To address this, we performed additional analyses using complementary MR-Egger, simple median and weighted median, each making different assumptions[41].

MR Steiger sensitivity testing was performed to test whether the assumption that the exposure causes outcome is valid[42]. This approach tests whether the genetic variants explain more of the variance in the outcome when the exposure is included in the model and, conversely, whether they explain more of the variance in the exposure when the outcome is included. If the genetic variants associated with the exposure also explain a significant portion of the variance in the outcome (and not vice versa), it provides supportive evidence for a causal relationship from exposure to outcome.

Multivariable MR was used to assess for independence of MR associations between cardiovascular traits as exposures and from key potential confounding factors as described in step 1[43]. Unlike univariable MR, which typically focuses on a single exposure variable, multivariable MR simultaneously considers several exposures to evaluate their collective impact on the outcome. This approach enables not only the direct MR associations of an exposure but can also highlight potential interactions. The outcome was regressed on genetic instruments for

both the exposure of interest and each confounding variable to determine the direct MR association of the exposure on the outcome after adjustment for a) each respective potential key confounding factor individually and b) all potential key confounding factors collectively in a multivariable MR model respectively.

In step 2, results are presented with one-tailed p-values to test the hypothesis that cardiovascular traits (CAD and stroke) have risk-increasing MR associations on IMID in Table 3. All other p-values in steps 1 and 3 are presented with two-tailed p-values as measures of statistical significance.

Study approval

Two-sample MR analyses do not involve human subjects, individual patient data, or any interaction or intervention with human subjects. Instead, two-sample MR exclusively uses genetic variants from previously published GWAS. As such, no ethical approval was required for these analyses.

The UK Biobank analyses were carried out using the UK Biobank Resource, approved under project number 15147. The UK Biobank holds approval from the Northwest Multi-centre Research Ethics Committee. It has also obtained clearance from the Patient Information Advisory Group in England and Wales to access data that enables the invitation of potential participants.

© 2024 Ramessur R et al. *JAMA Cardiology.*

eFigure 1

Schematic representation of bidirectional Mendelian Randomization (MR) analyses

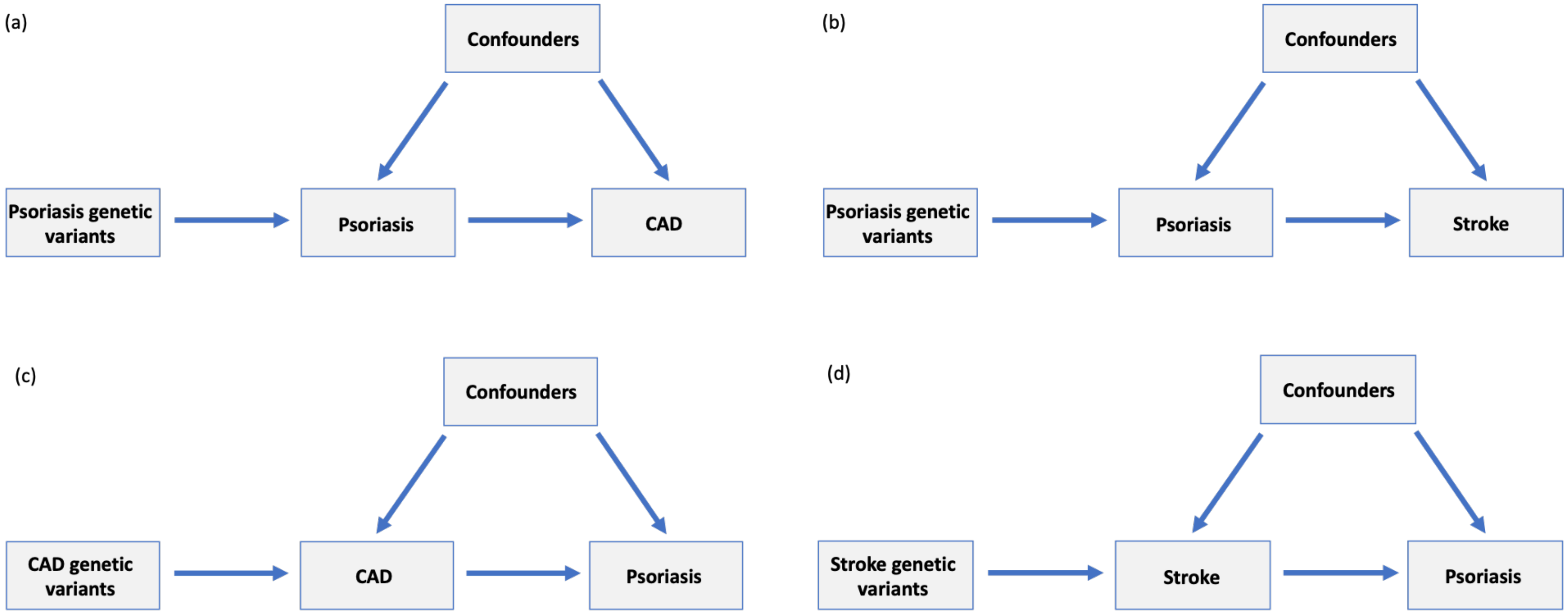

(a) Psoriasis genetic variants were used as instrumental variables to investigate the MR association of genetic predictors of psoriasis upon coronary artery disease (CAD). (b) Psoriasis genetic variants were also used as instrumental variables to investigate the MR association of genetic predictors of psoriasis upon stroke. (c) CAD genetic variants were used as instrumental variables to investigate the MR association of genetic predictors of CAD upon psoriasis. (d) Stroke genetic variants were used as instrumental variables to investigate the MR association of genetic predictors of stroke upon psoriasis. Arrows indicate MR assumption namely that the instrumental variable is associated with the exposure-not associated with confounders-and only affects the outcome via the exposure.

© 2024 Ramessur R et al. *JAMA Cardiology.*

Plots for two-sample Mendelian Randomization

eFigure 2a- Scatter plot for two-sample Mendelian Randomization (MR) analysis examining the effects of genetic predictors of coronary artery disease on psoriasis

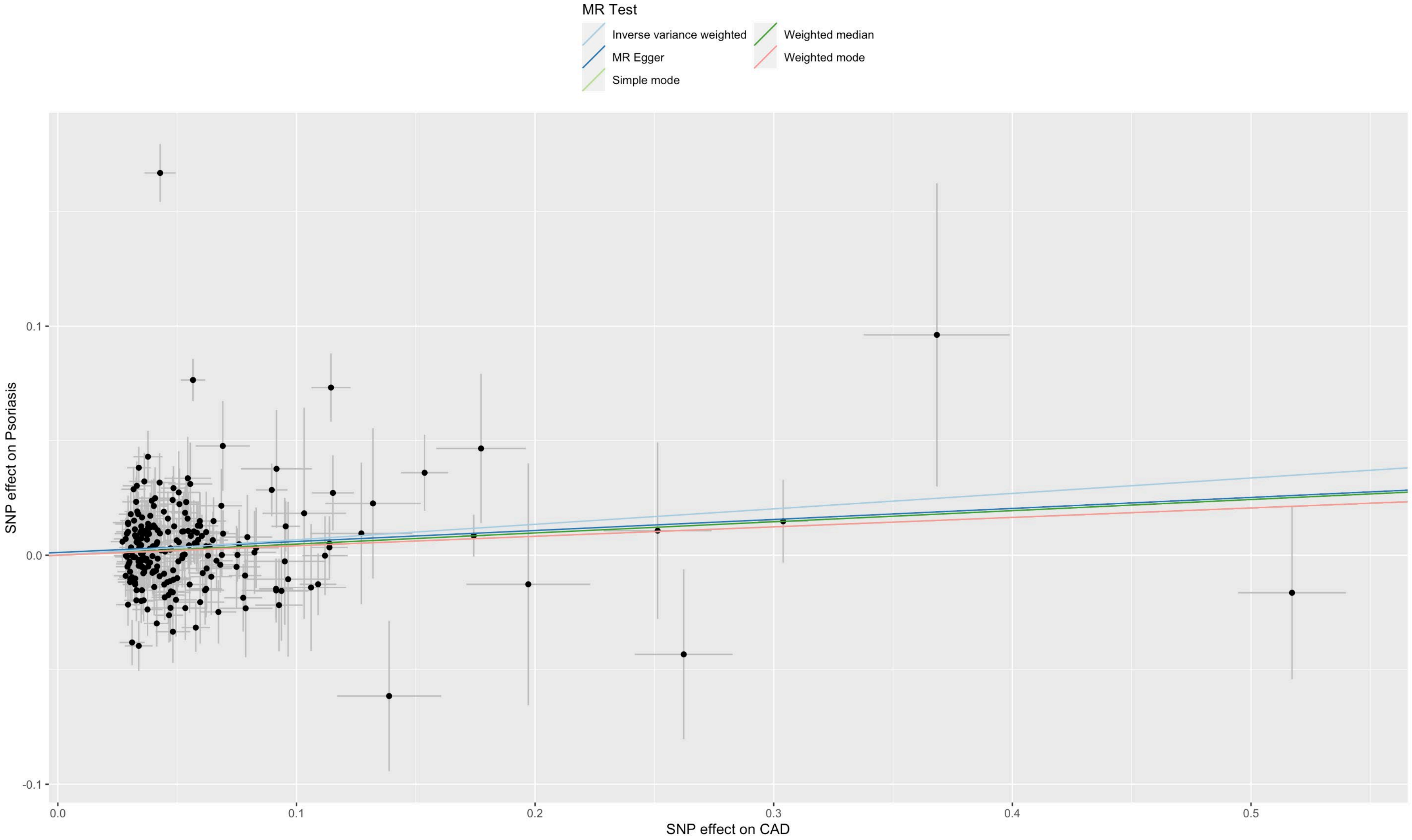

© 2024 Ramessur R et al. *JAMA Cardiology.*

eFigure 2b- Funnel plot for two-sample Mendelian Randomization (MR) analysis examining the effects of genetic predictors of coronary artery disease on psoriasis

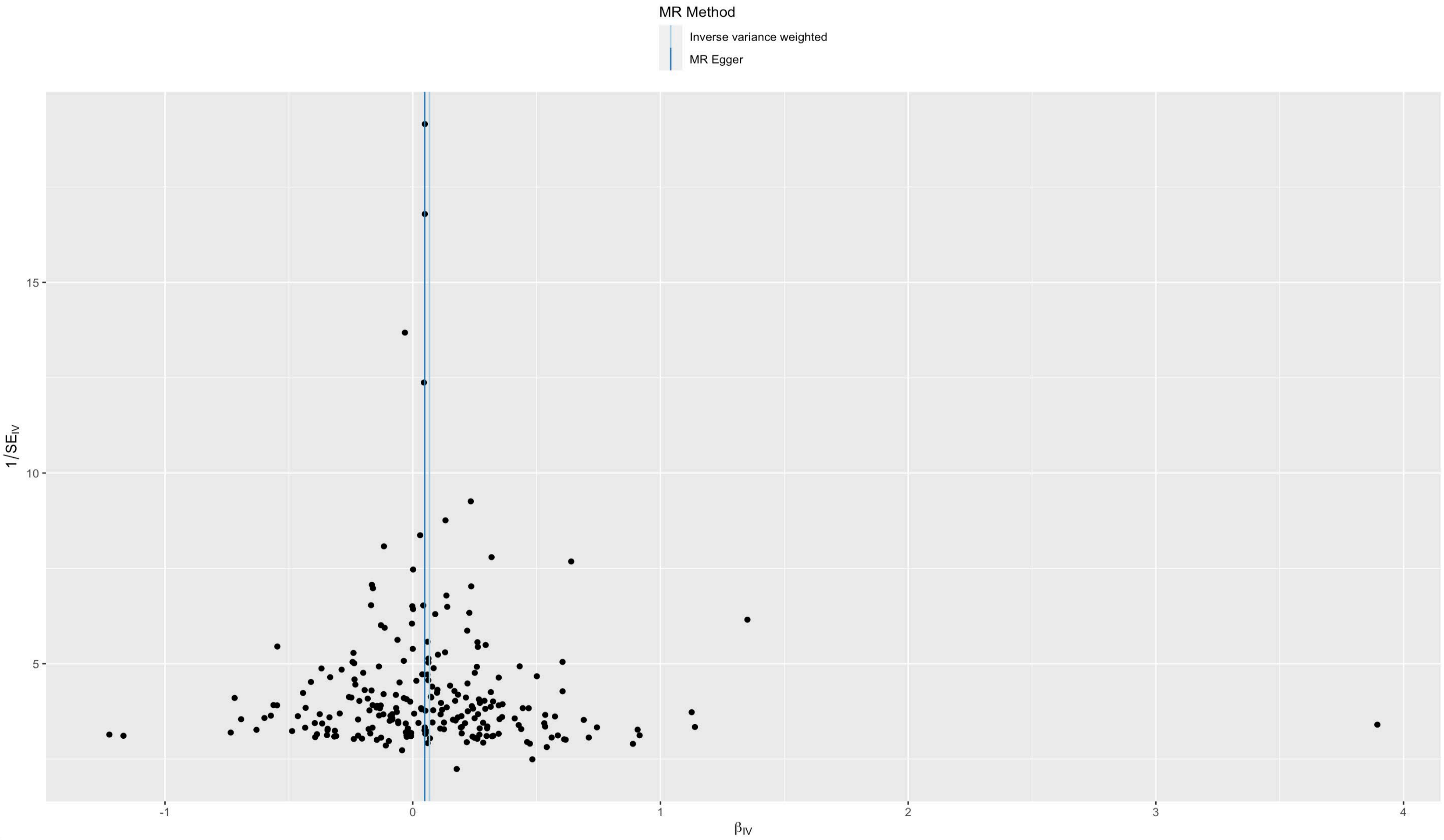

© 2024 Ramessur R et al. *JAMA Cardiology.*

eFigure 2c- Leave-one-out plot for two-sample Mendelian Randomization (MR) analysis examining the effects of genetic predictors of coronary artery disease on psoriasis

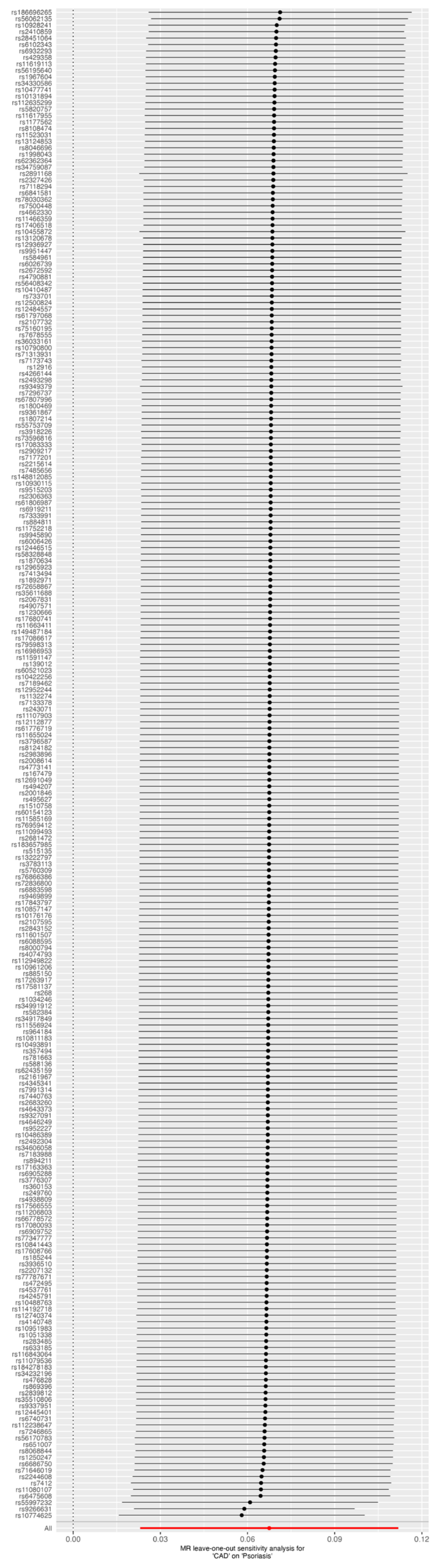

© 2024 Ramessur R et al. JAMA Cardiology.

eFigure 2d- Scatter plot for two-sample Mendelian Randomization (MR) analysis examining the effects of genetic predictors of stroke on psoriasis

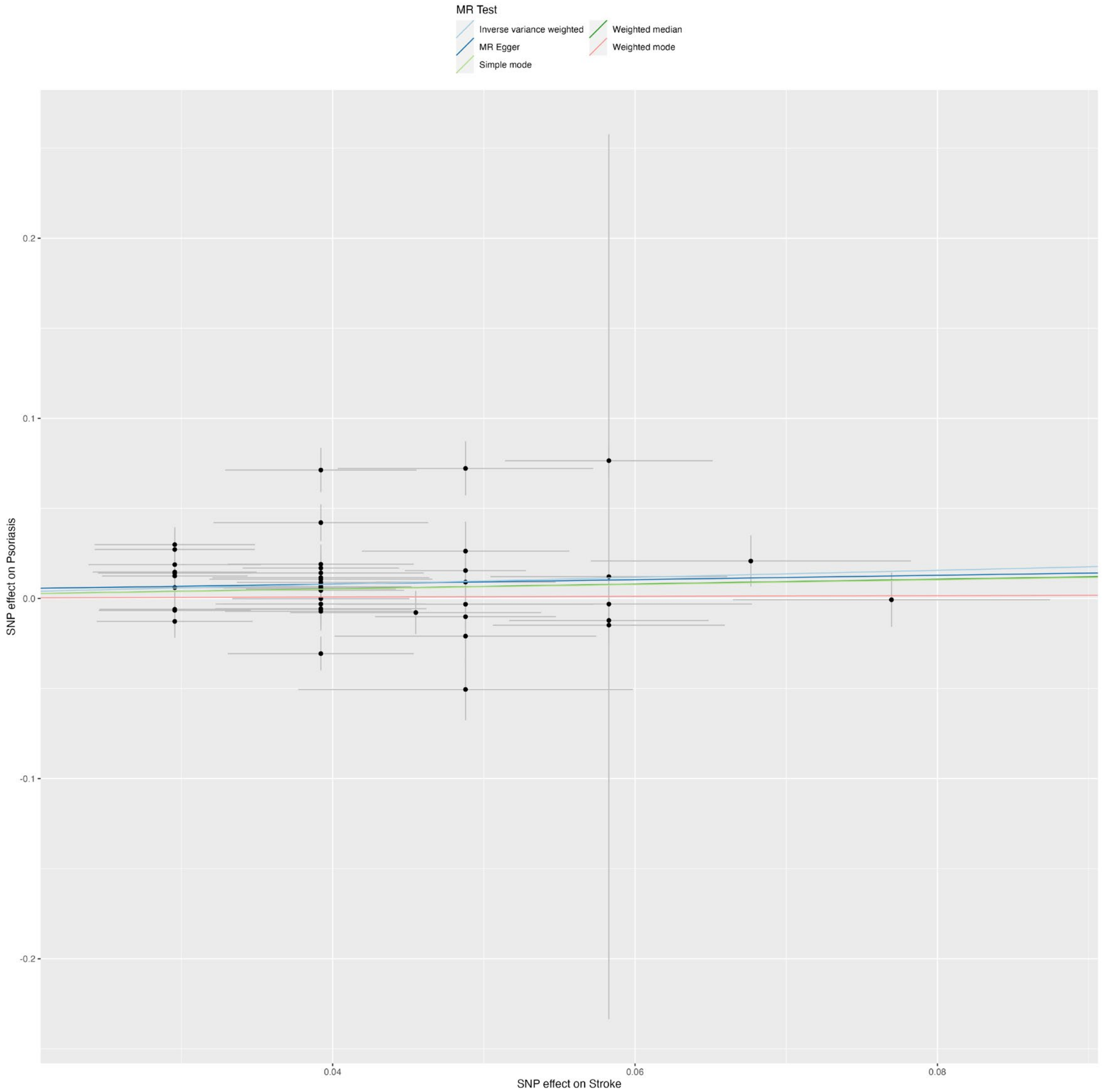

© 2024 Ramessur R et al. *JAMA Cardiology.*

eFigure 2e- Funnel plot for two-sample Mendelian Randomization (MR) analysis examining the effects of genetic predictors of stroke on psoriasis

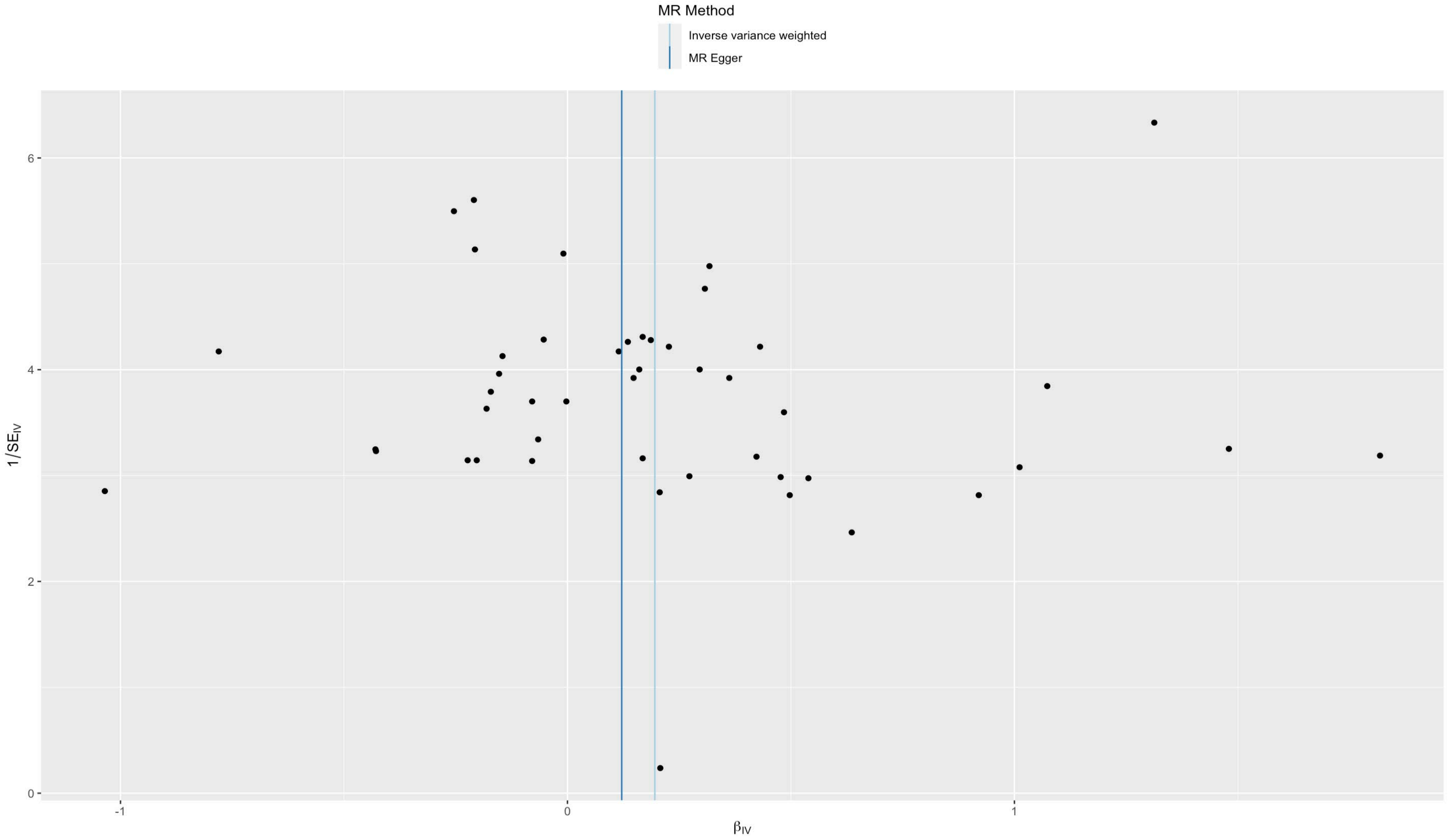

© 2024 Ramessur R et al. *JAMA Cardiology.*

eFigure 2f- Leave-one-out plot for two-sample Mendelian Randomization (MR) analysis examining the effects of genetic predictors of coronary artery disease on psoriasis

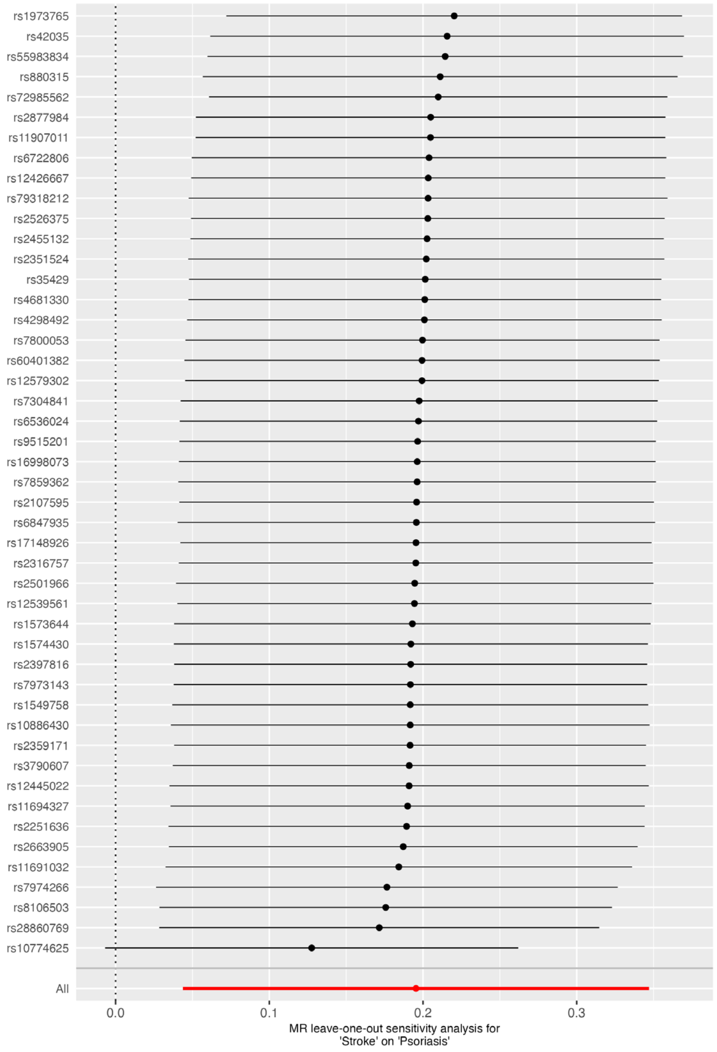

© 2024 Ramessur R et al. *JAMA Cardiology.*

eReferences

1. Burgess S, Davey Smith G, Davies NM, et al. Guidelines for performing Mendelian randomization investigations: update for summer 2023. *Wellcome Open Res*. 2019;4:186. doi:10.12688/wellcomeopenres.15555.3

2. Aragam KG, Jiang T, Goel A, et al. Discovery and systematic characterization of risk variants and genes for coronary artery disease in over a million participants. *Nat Genet*. Dec 2022;54(12):1803-1815. doi:10.1038/s41588-022-01233-6

3. Mishra A, Malik R, Hachiya T, et al. Stroke genetics informs drug discovery and risk prediction across ancestries. *Nature*. Nov 2022;611(7934):115-123. doi:10.1038/s41586-022-05165-3

4. Dand N, Stuart PE, Bowes J, et al. GWAS meta-analysis of psoriasis identifies new susceptibility alleles impacting disease mechanisms and therapeutic targets. *medRxiv*. Oct 5 2023;doi:10.1101/2023.10.04.23296543

5. Sinikumpu SP, Jokelainen J, Tasanen K, Huilaja L. Cardiovascular and Metabolic Profile of Subjects with Acne in a Cohort of Middle-aged Patients: A General Population Study of 1,932 Subjects. *Acta Derm Venereol*. Jul 10 2023;103:adv5921. doi:10.2340/actadv.v103.5921

6. Ascott A, Mulick A, Yu AM, et al. Atopic eczema and major cardiovascular outcomes: A systematic review and meta-analysis of population-based studies. *J Allergy Clin Immunol*. May 2019;143(5):1821-1829. doi:10.1016/j.jaci.2018.11.030

7. Zhang B, Li ZF, An ZY, et al. Association Between Asthma and All-Cause Mortality and Cardiovascular Disease Morbidity and Mortality: A Meta-Analysis of Cohort Studies. *Front Cardiovasc Med*. 2022;9:861798. doi:10.3389/fcvm.2022.861798

8. Emilsson L, Lebwohl B, Sundström J, Ludvigsson JF. Cardiovascular disease in patients with coeliac disease: A systematic review and meta-analysis. *Dig Liver Dis*. Oct 2015;47(10):847-52. doi:10.1016/j.dld.2015.06.004

9. Jaiswal V, Batra N, Dagar M, et al. Inflammatory bowel disease and associated cardiovascular disease outcomes: A systematic review. *Medicine (Baltimore)*. Feb 10 2023;102(6):e32775. doi:10.1097/md.0000000000032775

10. Rapp D, Michels S, Schöpe J, Schwingshackl L, Tumani H, Senel M. Associations between multiple sclerosis and incidence of heart diseases: Systematic review and meta-analysis of observational studies. *Mult Scler Relat Disord*. Nov 2021;56:103279. doi:10.1016/j.msard.2021.103279

11. Ogdie A, Yu Y, Haynes K, et al. Risk of major cardiovascular events in patients with psoriatic arthritis, psoriasis and rheumatoid arthritis: a population-based cohort study. *Annals of the Rheumatic Diseases*. 2015;74(2):326-332. doi:10.1136/annrheumdis-2014-205675

12. Sun HH, Tian F. Inflammatory bowel disease and cardiovascular disease incidence and mortality: A meta-analysis. *Eur J Prev Cardiol*. Oct 2018;25(15):1623-1631. doi:10.1177/2047487318792952

13. Garshick MS, Vaidean G, Nikain CA, Chen Y, Smilowitz NR, Berger JS. Sex differences in the prevalence of vascular disease and risk factors in young hospitalized patients with psoriasis. *Int J Womens Dermatol*. Sep 2019;5(4):251-255. doi:10.1016/j.ijwd.2019.05.003

14. Sondermann W, Djeudeu Deudjui DA, Körber A, et al. Psoriasis, cardiovascular risk factors and metabolic disorders: sex-specific findings of a population-based study. *J Eur Acad Dermatol Venereol*. Apr 2020;34(4):779-786. doi:10.1111/jdv.16029

15. Douroudis K, Ramessur R, Barbosa IA, et al. Differences in Clinical Features and Comorbid Burden between HLA-C∗06:02 Carrier Groups in >9,000 People with Psoriasis. *J Invest Dermatol*. Jun 2022;142(6):1617-1628.e10. doi:10.1016/j.jid.2021.08.446

© 2024 Ramessur R et al. *JAMA Cardiology.*

16. Locke AE, Kahali B, Berndt SI, et al. Genetic studies of body mass index yield new insights for obesity biology. *Nature*. Feb 12 2015;518(7538):197-206. doi:10.1038/nature14177

17. Liu M, Jiang Y, Wedow R, et al. Association studies of up to 1.2 million individuals yield new insights into the genetic etiology of tobacco and alcohol use. *Nat Genet*. Feb 2019;51(2):237-244. doi:10.1038/s41588-018-0307-5

18. Evangelou E, Warren HR, Mosen-Ansorena D, et al. Genetic analysis of over 1 million people identifies 535 new loci associated with blood pressure traits. *Nat Genet*. Oct 2018;50(10):1412-1425. doi:10.1038/s41588-018-0205-x

19. Wheeler E, Leong A, Liu CT, et al. Impact of common genetic determinants of Hemoglobin A1c on type 2 diabetes risk and diagnosis in ancestrally diverse populations: A transethnic genome-wide meta-analysis. *PLoS Med*. Sep 2017;14(9):e1002383. doi:10.1371/journal.pmed.1002383

20. Graham SE, Clarke SL, Wu KH, et al. The power of genetic diversity in genome-wide association studies of lipids. *Nature*. Dec 2021;600(7890):675-679. doi:10.1038/s41586-021-04064-3

21. Bycroft C, Freeman C, Petkova D, et al. The UK Biobank resource with deep phenotyping and genomic data. *Nature*. Oct 2018;562(7726):203-209. doi:10.1038/s41586-018-0579-z

22. Sudlow C, Gallacher J, Allen N, et al. UK biobank: an open access resource for identifying the causes of a wide range of complex diseases of middle and old age. *PLoS Med*. Mar 2015;12(3):e1001779. doi:10.1371/journal.pmed.1001779

23. Mitchell BL, Saklatvala JR, Dand N, et al. Genome-wide association meta-analysis identifies 29 new acne susceptibility loci. *Nature Communications*. 2022/02/07 2022;13(1):702. doi:10.1038/s41467-022-28252-5

24. Budu-Aggrey A, Kilanowski A, Sobczyk MK, et al. European and multi-ancestry genome-wide association meta-analysis of atopic dermatitis highlights importance of systemic immune regulation. *Nature Communications*. 2023/10/04 2023;14(1):6172. doi:10.1038/s41467-023-41180-2

25. Han Y, Jia Q, Jahani PS, et al. Genome-wide analysis highlights contribution of immune system pathways to the genetic architecture of asthma. *Nature Communications*. 2020/04/15 2020;11(1):1776. doi:10.1038/s41467-020-15649-3

26. Dubois PCA, Trynka G, Franke L, et al. Multiple common variants for celiac disease influencing immune gene expression. *Nature Genetics*. 2010/04/01 2010;42(4):295-302. doi:10.1038/ng.543

27. de Lange KM, Moutsianas L, Lee JC, et al. Genome-wide association study implicates immune activation of multiple integrin genes in inflammatory bowel disease. *Nature Genetics*. 2017/02/01 2017;49(2):256-261. doi:10.1038/ng.3760

28. Consortium*† IMSG, Patsopoulos NA, Baranzini SE, et al. Multiple sclerosis genomic map implicates peripheral immune cells and microglia in susceptibility. *Science*. 2019;365(6460):eaav7188. doi:doi:10.1126/science.aav7188

29. Okada Y, Wu D, Trynka G, et al. Genetics of rheumatoid arthritis contributes to biology and drug discovery. *Nature*. 2014/02/01 2014;506(7488):376-381. doi:10.1038/nature12873

30. Purcell S, Neale B, Todd-Brown K, et al. PLINK: a tool set for whole-genome association and population-based linkage analyses. *Am J Hum Genet*. Sep 2007;81(3):559-75. doi:10.1086/519795

31. Dilthey A, Leslie S, Moutsianas L, et al. Multi-population classical HLA type imputation. *PLoS Comput Biol*. 2013;9(2):e1002877. doi:10.1371/journal.pcbi.1002877

32. Kim GK, Del Rosso JQ. Drug-provoked psoriasis: is it drug induced or drug aggravated?: understanding pathophysiology and clinical relevance. *J Clin Aesthet Dermatol*. Jan 2010;3(1):32-8.

© 2024 Ramessur R et al. *JAMA Cardiology.*

33. Azzouz B, Abou Taam M, Morel A, Trenque T. Psoriasis during angiotensin receptor blocker exposure: an underestimated adverse drug reaction. *Expert Opin Drug Saf*. Sep 2018;17(9):853-857. doi:10.1080/14740338.2018.1508446

34. Cozzani E, Scaparro M, Parodi A. A case of psoriasis worsened by atorvastatin. *J Dermatol Case Rep*. Dec 30 2009;3(4):60-1. doi:10.3315/jdcr.2009.1037

35. Sanderson E, Spiller W, Bowden J. Testing and correcting for weak and pleiotropic instruments in two-sample multivariable Mendelian randomization. *Stat Med*. Nov 10 2021;40(25):5434-5452. doi:10.1002/sim.9133

36. Hemani G, Zheng J, Elsworth B, et al. The MR-Base platform supports systematic causal inference across the human phenome. *Elife*. May 30 2018;7doi:10.7554/eLife.34408

37. Burgess S, Thompson SG. Avoiding bias from weak instruments in Mendelian randomization studies. *Int J Epidemiol*. Jun 2011;40(3):755-64. doi:10.1093/ije/dyr036

38. Burgess S. Identifying the odds ratio estimated by a two-stage instrumental variable analysis with a logistic regression model. *Stat Med*. Nov 30 2013;32(27):4726-47. doi:10.1002/sim.5871

39. Burgess S, Small DS, Thompson SG. A review of instrumental variable estimators for Mendelian randomization. *Stat Methods Med Res*. Oct 2017;26(5):2333-2355. doi:10.1177/0962280215597579

40. Bowden J, Davey Smith G, Burgess S. Mendelian randomization with invalid instruments: effect estimation and bias detection through Egger regression. *Int J Epidemiol*. Apr 2015;44(2):512-25. doi:10.1093/ije/dyv080

41. Bowden J, Davey Smith G, Haycock PC, Burgess S. Consistent Estimation in Mendelian Randomization with Some Invalid Instruments Using a Weighted Median Estimator. *Genet Epidemiol*. May 2016;40(4):304-14. doi:10.1002/gepi.21965

42. Hemani G, Tilling K, Davey Smith G. Orienting the causal relationship between imprecisely measured traits using GWAS summary data. *PLoS Genet*. Nov 2017;13(11):e1007081. doi:10.1371/journal.pgen.1007081

43. Burgess S, Thompson SG. Multivariable Mendelian Randomization: The Use of Pleiotropic Genetic Variants to Estimate Causal Effects. *American Journal of Epidemiology*. 2015;181(4):251-260. doi:10.1093/aje/kwu283

© 2024 Ramessur R et al. *JAMA Cardiology.*
