# Supplementary material for: Exploring the Link Between Genetic Predictors of Cardiovascular Disease and Psoriasis
Source: JAMA Cardiol. 2024 Sep 18;9(11):1009–17. doi: 10.1001/jamacardio.2024.2859 (PMC11411451; doi:10.1001/jamacardio.2024.2859)
Supplement: Supplement 4. — Data Sharing Statement. [file jamacardiol-e242859-s004.pdf]

Data Sharing Statement

Ramessur. Exploring the Link Between Genetic Predictors of Cardiovascular Disease and Psoriasis. *JAMA Cardiol*. Published September 18, 2024. doi:10.1001/jamacardio.2024.2859

Data
Data available: No
